# Cytoskeletal Protein 4.1R in Health and Diseases

**DOI:** 10.3390/biom14020214

**Published:** 2024-02-11

**Authors:** Jiaojiao Liu, Cong Ding, Xin Liu, Qiaozhen Kang

**Affiliations:** 1 School of Life Science, Zhengzhou University, Zhengzhou 450001, China; ljj@gs.zzu.edu.cn; 2 Children’s Hospital Affiliated of Zhengzhou University, Zhengzhou 450018, China; dingcong8866@163.com

**Keywords:** 4.1R, *EPB41*, cytoskeletal protein, diseases, cancer

## Abstract

The protein 4.1R is an essential component of the erythrocyte membrane skeleton, serving as a key structural element and contributing to the regulation of the membrane’s physical properties, including mechanical stability and deformability, through its interaction with spectrin–actin. Recent research has uncovered additional roles of 4.1R beyond its function as a linker between the plasma membrane and the membrane skeleton. It has been found to play a crucial role in various biological processes, such as cell fate determination, cell cycle regulation, cell proliferation, and cell motility. Additionally, 4.1R has been implicated in cancer, with numerous studies demonstrating its potential as a diagnostic and prognostic biomarker for tumors. In this review, we provide an updated overview of the gene and protein structure of 4.1R, as well as its cellular functions in both physiological and pathological contexts.

## 1. Introduction

The red blood cell cytoskeleton is essential for maintaining the membrane’s physical characteristics, including mechanical stability and deformability. It is formed by a complex meshwork of proteins that imparts a great degree of elasticity. The red blood cell protein 4.1 is a crucial cytoskeletal protein found in mammalian erythrocytes. It plays a vital role in the mechanochemical properties of red blood cell membranes by facilitating the binding between spectrin and actin, and by aiding in the attachment of the membrane skeleton to the membrane [1]. Protein 4.1 was first discovered in human red blood cells. Its name derives from its identification as a specific band on an SDS gel of erythrocyte membranes (“band 4.1 protein”) [2]. An examination of erythrocytes from a patient with homozygous elliptocytosis revealed a lack of band 4.1 protein in the cell membranes. Band 4.1-deficient erythrocytes exhibited reduced mechanical strength and a marked tendency for fragmentation [3].

Band 4.1 protein, hereafter referred to as 4.1R (the prototypical protein found first in red blood cells), is one member of the protein 4.1 family—the others being 4.1N (neuronal type), 4.1G (general type), and 4.1B (brain type). The corresponding genes are erythrocyte membrane protein band 4.1 (*EPB41*), erythrocyte membrane protein band 4.1 like 1 (*EPB41L1*), erythrocyte membrane protein band 4.1 like 2 (*EPB41L2*), and erythrocyte membrane protein band 4.1 like 3 (*EPB41L3*) [4]. This review will be devoted to 4.1R and will update our knowledge of the gene structure and expression of 4.1R, as well as its protein structure and functions. Additionally, it will explore the roles of 4.1R in pathology and tumorigenesis.

## 2. Characteristics of 4.1R Gene, mRNA, and Protein

**Figure 1:**
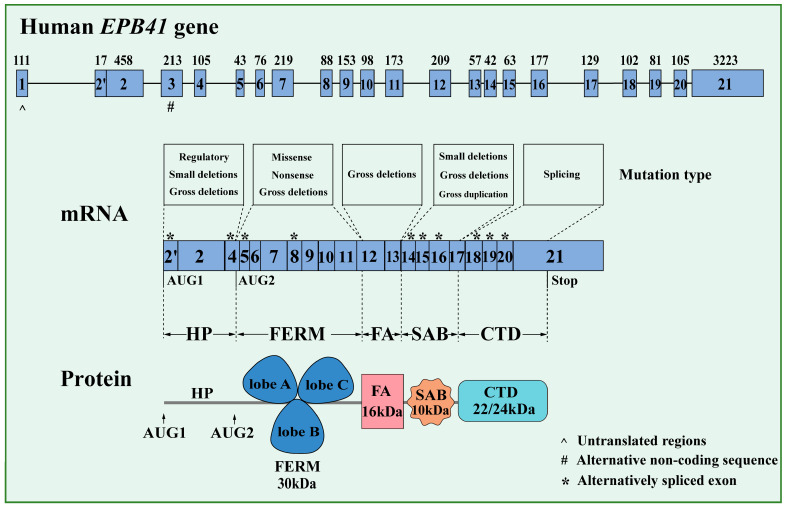
Gene, mRNA, and protein diagram of 4.1R. Mutation type: *EPB41* gene mutation associated with human disease. Protein domains: FERM—4.1 ezrin radixin moesin domain, FA—FERM-adjacent domain, SAB—spectrin–actin binding domain, and CTD—carboxyl terminal domain.

The human *EPB41* gene is located on chr1p35.3, while the mouse *EPB41* gene is located on chr4. A total of 21 exons code for the sequence of 4.1R. Four main functional domains have been identified in 4.1R (Figure 1). The N-terminal highly ordered 30 kDa globular membrane binding domain (MBD), known as a FERM (four-point-one ezrin radixin moesin) domain (amino acids 210–507), interacts with various proteins in erythroid and non-erythroid cells. It consists of three globular lobes (a “cloverleaf-like” structure), with distinct ligand binding capabilities in each lobe [5]. The N-terminal lobe (lobe A), which consists of the first 78 amino acids and 4 double-stranded β-strands, has a fold analogous to ubiquitin and binds to band 3 and the rhesus complex proteins Rh. The central lobe (lobe B), which consists of the next 90 amino acids, has an α-helical fold like acyl-CoA binding protein and binds to the transporter XK, the chemokine receptor Duffy, and glycophorin C (GPC). The C-terminal lobe (lobe C), which contains seven β-strands and ends with an α-helix, has a fold similar to a pleckstrin homology domain and binds to CD44, p55, and the phospholipid phosphatidylserine [6,7,8]. Phosphatidylinositol-4,5-biphosphate (PIP2) binds in a cleft between lobes A and C [9]. The overall folding structure of FERM domains is conserved, despite the level of sequence conservation being extremely low [4].

The second domain is the 16 kDa FERM-adjacent (FA) domain. This domain is phosphorylated by several protein kinases, including protein kinase C. Phosphorylation in this domain regulates interactions of both the FERM and spectrin–actin binding (SAB) domains [10]. 

The third domain, the 10 kDa SAB domain, strengthens the interaction between spectrin and actin and also binds to tropomyosin and myosin. The 21 amino acid peptides encoded by exon 16 in the SAB domain play an essential role in maintaining membrane stability by promoting spectrin/actin interactions [11].

The 22/24 kDa carboxyl-terminal domain (CTD) has been documented to exhibit binding affinity toward the immunophilin FKBP13 and the nuclear mitotic apparatus (NuMA) [12].

The *EPB41* pre-mRNA undergoes extensive alternative splicing, resulting in the production of multiple isoforms ranging from 30 to 210 kDa [13]. In mammals, the N-terminal of 4.1R has a variably spliced headpiece (also known as the U1 region, HP, amino acids 1–209). The HP region is an unstructured domain. Splicing of the HP region leads to the formation of the two most abundant isoforms of 4.1R, with apparent molecular masses of 135 kDa and 80 kDa on SDS gels [14]. The 4.1R^135^ isoform is exclusively expressed in early erythroblasts and other nucleated cells, while the 4.1R^80^ isoform is expressed during the late stages of erythroid differentiation and is the primary component of mature erythrocytes. Translation of 4.1R^80^ is initiated at AUG-2, located in exon 4, while translation of 4.1R^135^ is initiated at AUG-1, located in exon 2 [15]. The 4.1R^135^ isoform contains an additional HP region at the N-terminus compared to 4.1R^80^, and lacks a stretch of 21 amino acids for the interaction of 4.1R with spectrin [16]. Protein 4.1R^135^ has a theoretical molecular weight of ~100 kDa, and this difference is due to the unstructured nature of the HP region [17]. 

HP plays an important role in modulating the interaction of FERM with its two membrane binding partners, band 3 and GPC [16]. In comparison to the strong binding of 4.1R^135^ to band 3, its binding to GPC is notably weaker than that of 4.1R^80^. Another significant contrast between 4.1R^135^ and 4.1R^80^ is the calcium ion (Ca^2+^) dependence of their binding to calmodulin (CaM). The binding of CaM to 4.1R^80^ is Ca^2+^-independent, while the interaction of CaM with 4.1R^135^ is highly dependent on Ca^2+^. This distinction is directly linked to the HP region of 4.1R^135^ [16]. Furthermore, CaM significantly reduces the binding of 4.1R^135^ to band 3 in a Ca^2+^-dependent manner and eliminates its binding to GPC and p55. Unlike band 3 and GPC, which do not bind directly to the HP region, CaM binds to the HP region (a short polypeptide consisting of amino acids 70–80) in a Ca^2+^-dependent manner. Therefore, it is inferred that the CaM binding site located in the HP region serves as the primary binding site in 4.1R^135^, and this site prevents the interaction of CaM with the Ca^2+^-independent binding site in 4.1R^80^.

U2, which is located between the FERM domain and the SAB domain, is subject to variable splicing, as indicated by previous research [18]. Additionally, there is another variably spliced region, known as U3, situated between the SAB and CTD domains [19].

## 3. Function of 4.1R

**Figure 2:**
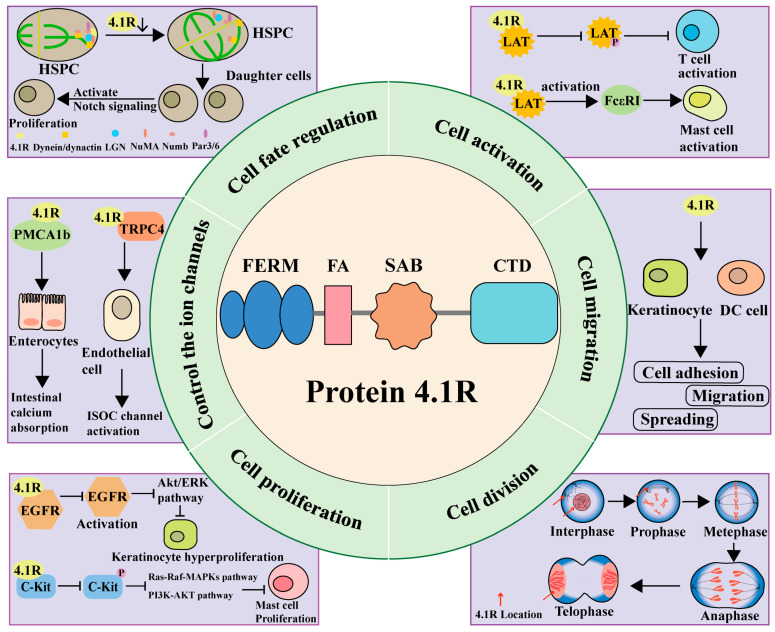
Structure and function of cytoskeleton protein 4.1R.

Recent studies have found that 4.1R serves not only as a connector between the plasma membrane and the membrane skeleton, but also interacts with numerous proteins and plays a crucial role in a variety of biological processes, including cell fate determination, cell cycle regulation, cell proliferation, and cell movement (Table 1; Figure 2).

### 3.1. Cell Fate Regulation

In B cells, 4.1R acts as a regulator of B-cell fate by inhibiting the canonical NF-κB signaling pathway, rather than non-canonical NF-κB regulating B-cell class switch recombination and plasma cell differentiation [24]. In addition, Huang et al. found a crucial function of 4.1R in the regulation of the unequal distribution of Numb and mediated the balance of fate in hematopoietic stem cells or erythropoiesis progenitor cells through its interaction with NuMA. Depletion of 4.1R resulted in the disconnection of LGN and NuMA from the cell cortex, leading to misorientation of the spindle. Despite this, dynein/dynactin can still be loaded onto the cell cortex through its direct interaction with Par3, allowing Numb to be transported to the cell cortex. However, Par3 alone is insufficient to target Numb in the cell cortex in the absence of 4.1R-LGN-NuMA, potentially leading to certain Numb components being unable to load into the cell cortex. Consequently, these depletions notably increase the proportion of symmetric distribution of Numb. Knockdown of 4.1R reduces the size of Numb in the daughter cells and enhances Notch signaling, directly promoting cell proliferation and delaying cell maturation [25].

### 3.2. Cell Activation

In T cells, 4.1R has been found to negatively regulate T-cell activation by directly interacting with LAT, leading to the inhibition of LAT phosphorylation and its downstream signaling molecule extracellular signal-regulated kinase (ERK) [28]. Additional investigations have demonstrated that 4.1R suppresses the activation of CD4^+^ T cells, thereby mitigating pathogenic autoimmunity in the progression of multiple sclerosis and experimental autoimmune encephalomyelitis progression [48]. Recent research has also indicated that 4.1R is a negative regulator of TCR signaling in CD8 T cells through its direct association with LAT [29]. In mast cells, Draberova et al. indicated that 4.1R acts as a positive regulator in the initial activation events following FcεRI triggering through its direct interaction with both LAT1 and LAT2 [27].

### 3.3. Cell Proliferation

A study by Ding et al. found that 4.1R inhibits mast cell proliferation by directly binding to the tyrosine kinase receptor C-Kit, thereby inhibiting C-Kit phosphorylation. It also negatively regulates the activation of the Ras-Raf-MAPKs and PI3K-AKT signal pathways [20]. In keratinocytes, a deficiency in 4.1R plays a role in sustaining abnormal EGFR-mediated cellular signaling and increasing the excessive proliferation potential of keratinocytes [32].

### 3.4. Cell Migration

Chen and colleagues observed a notable decrease in cell adhesion, spreading, migration, and motility in 4.1R-deficient keratinocytes, along with a reduction in the surface expression of β1 integrin. These findings indicate that 4.1R plays a functional role in keratinocytes by influencing the surface expression of β1 integrin through a direct interaction between 4.1R and β1 integrin [33]. In addition, Ruiz-Sáenz et al. indicated that 4.1R plays a crucial role in cell migration and the localization of the scaffold protein IQGAP1 to the leading edge of cells migrating into a wound [31]. The microtubule (MT) cytoskeleton is essential for cell polarity and migration. In a subsequent study, Ruiz-Saenz et al. showed that 4.1R associates with CLASP2 independently of MTs. They also found that 4.1R locally controls CLASP2 behavior, CLASP2 cortical platform turnover, and the organization, dynamics, and attachment of MTs to the cell cortex [37]. 

In addition, previous studies have found that there is an increased presence of 4.1R in the thymus of individuals with myasthenia gravis (MG). 4.1R may have a significant impact on the pathogenesis of MG in dendritic cells (DC). Silencing the expression of 4.1R led to a decrease in their ability to migrate, arrest in the cell cycle, and an increase in surface antigens in DC cells. This suggests that 4.1R plays a role in the autoimmune response in MG [49].

### 3.5. Control the Ion Channels

In endothelial cells, the interaction between 4.1R and TRPC4 is necessary for the activation of the store-operated calcium channel [30]. Liu showed that 4.1R^−/−^ mice displayed notable deficiencies in the absorption of calcium in the small intestine, as well as reduced levels of PMCA1b expression in enterocytes. These results indicate that 4.1R is directly related to PMCA1b, and the functional role of 4.1R in small intestine calcium absorption could be defined by regulating the membrane expression of PMCA1b [43].

### 3.6. Cell Division

As an adapter protein within nucleated cells, 4.1R is capable of integrating structural components of centrosome and is crucial for ensuring the fidelity of centrosome function [50]. 4.1R is rearranged during cell division [51]. It is located within the nucleus and centrosomes of cells during the interphase stage and rapidly redistributes to the developing spindle poles when the nuclear envelope disassembles in prometaphase. Additionally, it is detected in the perichromatin during telophase and in the midbody during cytokinesis [52,53]. These results indicate that 4.1R may have a substantial impact on nuclear structure and ultimately influence nuclear function. Initially, Mattagajasingh et al. discovered that 4.1R was linked to the spindle pole protein NuMA within the interphase nucleus. During the process of cell division, it also interacts with spindle pole organizing proteins, NuMA, dynein, and dynactin to form a complex. 4.1R may have a significant impact on the organization of nuclear architecture, mitotic spindle, and spindle poles [26]. Subsequently, Huang et al. proposed the significance of a 135-kDa non-erythroid 4.1R protein in cellular division. This protein is involved in the assembly of mitotic spindles and spindle poles by interacting with mitotic microtubules [54]. Krauss and colleagues demonstrated that immunodepletion of 4.1R disrupted microtubule arrays and mislocalized NuMA. They identified two 4.1R domains critical for its function: the SAB domain and the NuMA binding C-terminal domain [55]. Downregulation of 4.1R affected cell cycle progression and caused abnormalities in mitotic spindles and anaphase. Their findings provided functional evidence supporting the significant role of 4.1R in maintaining the structural integrity of centrosomes and mitotic spindles [56]. Mattagajasingh et al. identified the amino acids of 4.1R and NuMA that sustained their interaction. They demonstrated that the inhibition of the interaction between the protein 4.1R and NuMA through mutagenization of their binding sites resulted in the abrogation of nuclear localization of 4.1R [57]. Meyer and colleagues presented proof that 4.1R has significant functional interactions with the nuclear envelope protein emerin and the intermediate filament protein lamin A. These connections affect the nuclear structure, the association between the centrosome and nuclear envelope, and the regulation of β-catenin transcriptional co-activator activity, which relies on β-catenin nuclear export [58]. Recently, Huang et al. suggested that 4.1R regulates the asymmetric segregation of the Notch signaling regulatory protein Numb during terminal erythroid maturation. They also identified a critical role for 4.1R in mediating erythropoiesis [25].

## 4. 4.1R and Disease

**Figure 3:**
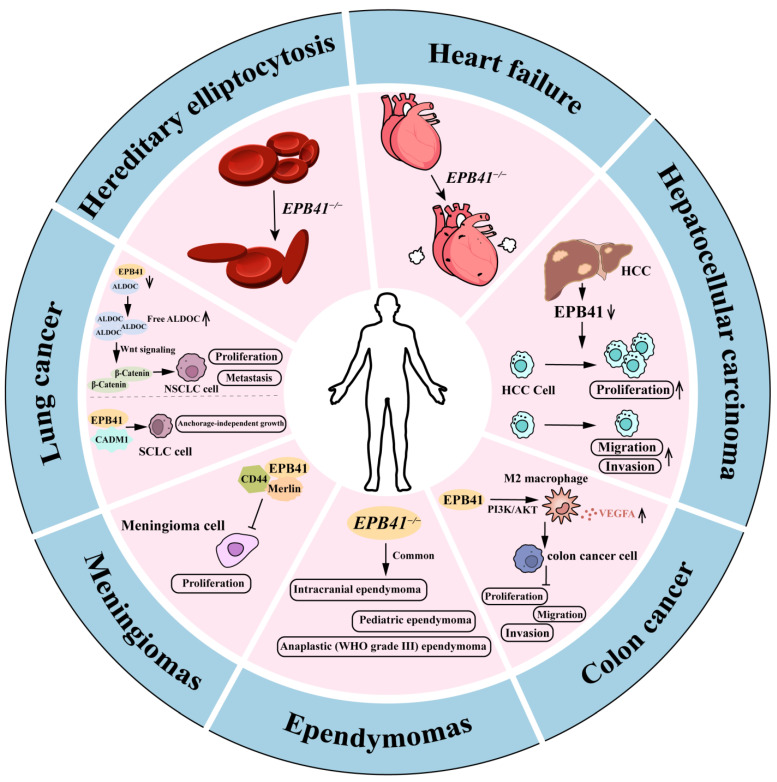
Overview of 4.1R and human disease. The figure shows the dysfunction of 4.1R in HE, HF, and tumors. The direction of the arrows representing rising or falling.

*EPB41* gene mutations, as well as a quantitative deficiency of protein 4.1R or defective assembly of structurally altered protein 4.1R, can lead to human disease (Table 2; Figure 3). 

### 4.1. Hereditary Elliptocytosis (HE)

HE, also referred to as hereditary ovalocytosis, is a genetically heterogeneous blood disorder characterized by oval-shaped erythrocytes with variable levels of hemolytic anemia [73]. HE is distributed globally, with a higher incidence in regions endemic to malaria, particularly among individuals of African and Mediterranean descent [74]. The actual prevalence of HE remains uncertain due to the variability in its clinical severity and the presence of many asymptomatic patients. Nevertheless, symptomatic patients should receive treatment involving blood transfusion and splenectomy [75]. Elliptocytosis is typically inherited as an autosomal dominant trait and is caused by mutations in various genes encoding proteins of the red blood cell cytoskeleton. The instability of membrane skeletons, cell membranes, and erythrocytes can lead to the fragmentation and hemolysis of red blood cells [76]. The primary cause of HE-associated lesions is attributed to qualitative and quantitative defects in the red blood cell membrane skeleton proteins, such as alpha spectrin, beta spectrin, protein 4.1R, band 3, and rarely, GPC [77,78,79]. 

Heterozygous or homozygous mutation of the *EPB41* gene causes HE-1 [80]. A partial deficiency in protein 4.1R has been linked to mild, dominant HE, whereas a complete deficiency is associated with severe hemolytic disease [61,81]. In a family from Algeria, people with HE exhibited severe hemolytic anemia due to protein 4.1R deficiency. The mutant gene had a DNA rearrangement from the upstream translation initiation codon. Additionally, the mRNA from the mutant gene was spliced in an abnormal manner [82]. Variants of protein 4.1R with abnormal molecular weights have also been observed in individuals with HE, predominantly by deletions or duplications of the exons located around the SAB domain [70,83]. A higher molecular weight form of 4.1R (95 kDa) is related to mild elliptocytosis without anemia, whereas a lower molecular weight form (65/68 kDa) is associated with moderate elliptocytosis and anemia. Erythrocyte membranes containing 4.1R (95 kDa) showed similar mechanical stability to normal membranes, consistent with the presence of an intact SAB domain in protein 4.1R. On the contrary, membranes containing 4.1R (65/68 kDa) displayed significantly reduced mechanical stability due to the absence of the SAB domain [84].

The constitutional deficit of protein 4.1R has been linked to HE. Acquired deficits of 4.1R have been reported in myelodysplastic syndromes with elliptocytosis [85]. Eight cases of acquired elliptocytosis have already been reported in myelodysplastic syndrome (MDS) or chronic myeloproliferative disorders (CMPD) [86,87,88]. Alanio-Bréchot et al. reported six patients with MDS or CMPDs and an acquired CMPD who had a deficiency in protein 4.1R. Alanio-Bréchot et al. confirmed that a deficiency in protein 4.1R is a recurring occurrence in myeloid malignancies when the deletion del (20q) is present. They discovered this chromosomal abnormality in four out of six patients. They found that the expression of the *EPB41* gene, rather than its structure, was disturbed due to the malignancy [89].

### 4.2. HF

HF is a prominent contributor to mortality in cardiovascular diseases worldwide, characterized by intricate clinical symptoms and a high rate of mortality [90]. Research has indicated that the myocardial cytoskeleton plays an important role by stabilizing the myocardium, detecting mechanical stretching, and coordinating cellular organization and intercellular signaling [91]. 4.1R is expressed in the heart, but its functional role in the myocardium is unknown. Stagg and colleagues published the initial findings of a cardiac phenotype linked to the absence of 4.1R [92]. In this study, Stagg et al. reported that 4.1R^−/−^ mice displayed a reduced heart rate along with a prolonged Q-T interval. The isolated 4.1R^−/−^ ventricular cardiomyocytes displayed prolonged action potentials, aberrant Ca^2+^ transients, increased sarcoplasmic reticulum Ca^2+^ stores, and increased spark frequency. The data indicated that 4.1R plays an unexpected role in regulating the functional properties of several cardiac ion transporters, thereby influencing cardiac electrophysiology. This suggests that 4.1R may have an important role in both normal heart function and disease. Then, Pinder et al. characterized the expression, distribution, and novel activities of 4.1R in the left ventricle. They detected an 80 kDa isoform of 4.1R in subcellular fractions that were enriched in intercalated discs. The presence of 4.1R overlapped with the plasma membrane signaling proteins, including the Na/K-ATPase and the Na/Ca exchanger NCX1, at the intercalated disc [93]. 

Wei and colleagues assessed the expression of 4.1R in cardiomyocytes and determined its potential role in the development of HF. Their findings revealed a significantly higher proportion of 4.1R-positive cells in the HF group compared to the control group. Their study also observed that 4.1R was primarily localized to the plasma membrane of myocardial cells and was upregulated as HF progressed [94]. Recently, Ning et al. detected co-localization and interaction between 4.1R and Nav1.5. These results suggest that 4.1R may play a role in the occurrence and progression of HF by interacting with ion channel proteins [41]. These findings suggest that there may be an association between 4.1R and the progression of HF, making it a promising therapeutic target for HF.

### 4.3. Tumors

4.1R exhibits various expressions and functions across different types of tumors. It is a potential marker for tumor prognosis and a target for tumor treatment.

#### 4.3.1. The Role of 4.1R in Tumors

A study by Yang et al. reported that there is a decreased expression of 4.1R in HCC tissues compared to adjacent normal tissues. Furthermore, the study found that 4.1R can significantly suppress the growth and progression of HCC [65]. 

Yuan et al. indicated that *EPB41* is a novel tumor suppressor in NSCLC. Their study demonstrated that the inhibition of *EPB41* expression in cancer cells increased the levels of ALDOC protein released from the EPB41-ALDOC complex. This, in turn, resulted in the upregulation of multiple oncogenes through the β-Catenin/TCF/LEF TF complex, leading to the pathogenesis of NSCLC [46].

SCLC is another malignancy with high expression of CADM1, leading to increased malignant characteristics. Funaki et al. found that 4.1R was necessary for the oncogenic effect of CADM1 in SCLC. CADM1 expression was observed to correlate with the membrane localization of 4.1R in both SCLC primary and cell lines. Additionally, the co-localization of CADM1 and 4.1R on the cell membrane was associated with a more advanced tumor stage. These findings indicate that the formation of the CADM1-4.1R complex contributes to the malignant features of SCLC [35]. Further investigation is needed to elucidate the mechanism of the CADM1-4.1R complex in the development and progression of SCLC.

Meningiomas are prevalent neoplasms of the central nervous system (CNS). One of the most common events associated with meningioma tumorigenesis is the deletion of chromosome 22q and the inactivation of the neurofibromatosis 2 gene [95,96]. Robb et al. observed a loss of 4.1R expression in 2 meningioma cell lines (IOMM-Lee and CH157-MN) as well as in 6 of 15 sporadic meningiomas. They demonstrated that 4.1R was a tumor suppressor in the molecular pathogenesis of meningioma [45]. However, Piaskowski et al. observed that the expression of 4.1R mRNA was unchanged in all analyzed meningiomas and suggested that the role of 4.1R in meningioma development should be reconsidered [97].

Ependymomas are prevalent malignant tumors affecting both pediatric and adult CNSs. Rajaram et al. found that losses of 4.1R expression and 4.1B (18p11.3) deletions were more frequently observed in pediatric, intracranial, and/or anaplastic (WHO grade III) ependymoma subtypes. Furthermore, the deletion of 4.1G (6q23) was linked to a more aggressive clinical disease. The researchers determined that alterations in protein 4.1 family members were widespread in ependymal tumors, and specific alterations were linked to distinct clinicopathologic subsets [98].

Li et al. revealed that protein 4.1R interacts with GAT-1 and GAT-2, leading to an impact on the transmembrane transport of 5-ALA. This interaction results in a decreased sensitivity of B16 cells to photodynamic therapy (PDT) and downregulates the anti-tumor immune response triggered by PDT [21].

M2 macrophages play a critical role in the tumor microenvironment and have been demonstrated to be closely associated with tumor progression. Lu et al. reported that 4.1R downregulated the secretion of vascular endothelial growth factor A (VEGFA) in M2 macrophages, thereby delaying colon cancer progression through the inhibition of the PI3K/AKT signaling pathway [99].

#### 4.3.2. Prognostic Marker of Disease

Individuals who receive a diagnosis of cancer at earlier or intermediate stages generally have more favorable prognoses compared to those diagnosed with advanced disease. So, early prediction and intervention represent the most efficacious approaches for enhancing the clinical outcomes of patients [100]. In recent years, numerous reports have confirmed that the expression of 4.1R is altered in various malignancies, either decreased or increased. Abnormal expression of 4.1R has been found to contribute to tumorigenesis and tumor progression. Feng et al. found that high expressions of 4.1R mRNA were associated with better survival in breast cancer patients. They concluded that 4.1R can be considered a novel biomarker and a potential therapeutic target for breast cancer [101]. In addition, it was found that the expression of 4.1R was weak in NSCLC tissues compared to normal tissues. Low expression of 4.1R was associated with a poor prognosis for lung cancer patients [46,102,103]. Liu et al. identified a two-gene (*PML-EPB41*) signature as a prognostic predictor for patients with osteosarcoma. This signature was validated and analyzed by an external dataset and biological experiment [104]. Yin et al. identified *EPB41* as a prognostic risk biomarker (PRB) with high potential as a drug target for the treatment of colon adenocarcinoma [105].

These studies elucidated the potential of *EPB41* as a future therapeutic target for cancer.

## 5. Conclusions and Future Perspectives

In this review, we summarize the structural characteristics, physiological functions, and pathological functions of 4.1R. As a structural protein, the function of 4.1R in mature erythrocytes has been extensively studied. In recent years, many studies have been carried out on its function in nucleated cells. 4.1R is involved in many cellular processes by regulating the cytoskeleton and signaling pathways. Any interference with the expression of 4.1R leads to disruptions in normal cell function and pathological outcomes. 

Genetic mutations associated with human disease have been reported to occur in different domains of the protein 4.1R, but many details remain unknown. In order to better understand the relationship between the structure and function of 4.1R and human diseases, the regulatory functions and mechanisms of different 4.1R domains in human diseases need to be further studied. Multiple isoforms of protein 4.1R are expressed in various tissues through complex pre-mRNA splicing events. During cell differentiation, the expression and localization of the *EPB41* gene are regulated. Therefore, the regulatory mechanisms of different subtypes or multiple subtypes of 4.1R in different diseases still need to be further studied. Elucidation of the regulatory functions and molecular mechanisms of 4.1R in diseases will provide a foundation for clinical diagnosis and personalized gene therapy for patients with *EPB41* mutations or expression deficiencies.

## Figures and Tables

**Table 1:** Binding proteins of 4.1R.

Binding Proteins	Mechanism/Pathway	Function	Binding Domain	Detection Method	Reference
C-Kit	Ras-Raf-MAPKs and PI3K-AKT	Negatively regulates mast cell proliferation.	FERM	Co-immunoprecipitation and pull-down	[20]
GAT-1/GAT-2	—	Affects photodynamic therapy for B16 melanoma and	—	Immunofluorescence and co-immunoprecipitation	[21]
	—	affects the transport of 5-aminolevulinic acid into mouse embryonic fibroblast cells.	—	Co-immunoprecipitation	[22]
von Hippel–Lindau (VHL)	Reverses myogenin ubiquitination and stability	Influences myogenesis.	—	Immunofluorescence and co-immunoprecipitation	[23]
Toll-like receptor 4 (TLR4)	Canonical nuclear factor kappa-B (NF-κB) pathway	Modulates B-cell fate.	—	Immunofluorescence and co-immunoprecipitation	[24]
NuMA	Notch signaling	Regulates the asymmetric segregation of the Numb to mediate erythropoiesis.	CTD	co-immunoprecipitation	[25]
	4.1R alters the normal distribution of NuMA in the interphase nucleus	Organizes the nuclear architecture, mitotic spindle, and spindle poles.	CTD	Yeast two-hybrid assay, immunofluorescence, co-immunoprecipitation, and pull-down	[26]
Linker for activation of T cells (LAT)1/LAT2	FcεRI Signaling	Acts as a positive regulator in the early activation events in mast cells.	—	Co-immunoprecipitation	[27]
LAT	4.1R negatively regulates signaling from the T-cell antigen receptor (TCR) through LAT to the MAP kinase pathway	Negatively regulates T-cell activation.	FERM	Co-immunoprecipitation and pull-down	[28]
	TCR-mediated signal transduction	Negatively regulates CD8 T-cell activation.	—	Co-immunoprecipitation	[29]
Transient receptor potential canonical 4 (TRPC4)	4.1R interacts with TRPC4 and the membrane skeleton	Activates the endothelial *I*_SOC_ channel.	—	Co-immunoprecipitation	[30]
IQ motif-containing GTPase-activating protein 1 (IQGAP1)	4.1R is necessary for the localization of IQGAP1 to the leading edge of cells	Cell migration and the recruitment of the scaffold protein IQGAP1 to the cell front.	FERM	Immunofluorescence, co-immunoprecipitation, and pull-down	[31]
Epidermal growth factor receptor (EGFR)	Akt/ERK signaling	Regulation of EGFR activation and EGFR signaling in keratinocytes.	—	Immunofluorescence and co-immunoprecipitation	[32]
β1 integrin	Modulating surface expression of beta1 integrin	Regulates cell adhesion, spreading, migration, and motility of mouse keratinocytes.	FERM	Co-immunoprecipitation and pull-down	[33]
β-catenin	Linking the cadherin/catenin complex to the cytoskeleton through its direct interaction with β-catenin	Regulates the integrity of adherens junction in the gastric epithelial cells.	FERM	Immunofluorescence, co-immunoprecipitation, and pull-down	[34]
Cell adhesion molecule 1 (CADM1)	Membranous co-localization of CADM1 and 4.1R	Promotes malignant features of small cell lung cancer (SCLC).	—	Immunofluorescence, co-immunoprecipitation, and immunohistochemistry	[35]
Eukaryotic translation initiation factor 3 (elF3-p44)	4.1R direct association with elF3-p44	An anchor protein that links the cytoskeleton network to the translation apparatus.	CTD	Yeast two-hybrid assay, co-immunoprecipitation, and pull-down	[36]
Cytoplasmic linker-associated protein-2 (CLASP2)	4.1R controls CLASP2 behavior, CLASP2 cortical platform turnover, and GSK3 activity	Correct MT organization and dynamics essential for cell polarity.	FERM	Immunofluorescence, co-immunoprecipitation, and pull-down	[37]
ICln	Affecting 4.1R interaction with β-actin	Cell volume regulation and cell morphology.	FERM	Yeast two-hybrid assay, co-immunoprecipitation, pull-down, and fluorescence resonance energy transfer (FRET)	[38,39]
ZO-2	The link between the tight junction and the actin cytoskeleton	Organizes the tight junction.	CTD	Yeast two-hybrid assay, immunofluorescence, co-immunoprecipitation, and pull-down	[40]
Voltage-gated Sodium Channel 1.5 (NaV1.5)	4.1R interaction with ion channel proteins	Involved in the occurrence and development of heart failure (HF).	—	Immunofluorescence and co-immunoprecipitation	[41]
Metabotropic glutamate receptor type 8 (mGluR8)	mGluR8-mediated signal transduction	Correct regulation of neurotransmitter receptors.	CTD	Yeast two-hybrid assay	[42]
Plasma membrane calcium ATPase 1b (PMCA1b)	Regulation of membrane expression of PMCA1b	Regulates intestinal Ca^2+^ absorption.	FERM	Immunofluorescence, co-immunoprecipitation, and pull-down	[43]
Human discs large isoform (hDlg-I_3_)	—	Recruits hDlg to the lateral membrane in polarized epithelial cells.	FERM	Pull-down	[44]
CD44	—	Acts as an important tumor suppressor in the molecular pathogenesis of meningioma.	FERM	Co-immunoprecipitation	[45]
Aldolase C (ALDOC)	Wnt signaling	Inhibits non-small cell lung cancer (NSCLC) proliferation, invasion, and metastasis in vitro and in vivo.	—	Immunofluorescence and co-immunoprecipitation	[46]
Centrosomal P4.1-associated protein (CPAP)	—	Cell division and centrosome function.	HP	Yeast two-hybrid assay, co-immunoprecipitation, and pull-down	[47]

**Table 2:** All published *EPB41* gene lesions responsible for human inherited disease. Data from the Human Gene Mutation Database (HGMD) [59].

Mutation Type	Mutation Data by Type			Mutation Position	Phenotype	Reference
**Missense/nonsense**	**Codon change**	**Amino acid change**	**Codon number**			
	AUG-ACG	Met-Thr	1	AUG2	Elliptocytosis	[60]
	AUG-AGG	Met-Arg	1	AUG2	Elliptocytosis	[61]
	TAT-TAA	Tyr-Ter	233	FERM	Hemolytic anemia	[62]
	CGA-TGA	Arg-Ter	262	FERM	Elliptocytosis	[63]
	ACA-ATA	Thr-Ile	283	FERM	Spherocytosis	[64]
**Splicing**	**Splicing mutation**					
	a base substitution at position 2720 (G→A)			CTD	Elliptocytosis	[12]
**Regulatory**	**Sequence**					
	−278 relative to transcription initiation site			HP	Hepatocellular carcinoma (HCC), increased risk	[65]
**Small deletions**	**Deletion**					
	GAATCAG			HP	Elliptocytosis	[66]
	A			SAB	Elliptocytosis	[67]
	AAA			SAB	Elliptocytosis	[68]
**Gross deletions**	**Description**					
	exons 2 to 12			HP, FERM, and FA	Elliptocytosis	[69]
	240 bp (Lys407-Gly486)			SAB	Elliptocytosis	[70]
	318 bp			HP and FERM	Elliptocytosis	[71]
	50 kb of genomic DNA, exons 2 and 4			HP	Elliptocytosis	[72]
**Gross duplication**	**Description**					
	369 bp (Lys407-Gln529)			SAB	Elliptocytosis	[70]
